# Combining deterministic and probabilistic matching to reduce data linkage errors in hospital administrative data

**DOI:** 10.23889/ijpds.v1i1.316

**Published:** 2017-04-19

**Authors:** Gareth Hagger-Johnson, Katie Harron, Rob Aldridge, Bo Fu, Efrosini Setakis, Harvey Goldstein, Ruth Gilbert

**Affiliations:** Administrative Data Research Centre England (ADRC-E), University College London; London School of Hygiene & Tropical Medicine; University College London; University of Bristol

## Objectives

Data linkage algorithms are used to link together multiple episodes of care belonging to the same patient. For example, the HESID algorithm is used to generate Hospital Episode Statistics (HES) in England. HESID is a deterministic algorithm, requiring identifiers to agree or disagree at each step. Data linkage errors occur when episodes belonging to two patients are incorrectly linked (a false match) or when episodes belonging to the same patient are not linked (a missed match). This typically occurs because patient identifiers (e.g. NHS number, postcode) contain errors or have missing data. We previously showed that HESID has a low false match rate (0.2%) but a high missed match rate (4.1%) when applied to paediatric intensive care data. This biased the true readmission rate, particularly for some patient groups including ethnic minorities. The aim of our study was to evaluate whether an additional step involving probabilistic matching would lower the missed match rate in HES without increasing the false matched rate. 

## Approach

We simulated three datasets having the same characteristics as HES, for three age groups expected to have different levels of postcode stability (at age 0/1, 5/6 and 18/19). We compared the deterministic algorithm to a probabilistic algorithm, and then to a deterministic algorithm with an additional probabilistic step. In sensitivity analyses, we evaluated the algorithms under different data quality scenarios. 

## Results

Results show that deterministic followed by probabilistic matching is the best solution for reducing missed matches, particularly in scenarios where errors in patient identifiers are more common.

## Conclusions

Data linkage algorithms need to be evaluated against good quality reference standard data sets. For hospital data in England, the Personal Demographics Service (PDS) could be used to evaluate our approach, because it contains many of the same patient identifiers used in HES. Reducing data linkage error will improve monitoring of hospital activity in England.

